# Integrative analysis reveals novel insights into juvenile idiopathic arthritis pathogenesis and shared molecular pathways with associated traits

**DOI:** 10.3389/fgene.2024.1448363

**Published:** 2024-08-08

**Authors:** N. Pudjihartono, D. Ho, J. M. O’Sullivan

**Affiliations:** ^1^ The Liggins Institute, The University of Auckland, Auckland, New Zealand; ^2^ The Maurice Wilkins Centre, The University of Auckland, Auckland, New Zealand; ^3^ MRC Lifecourse Epidemiology Unit, University of Southampton, Southampton, United Kingdom; ^4^ Australian Parkinsons Mission, Garvan Institute of Medical Research, Sydney, NSW, Australia; ^5^ A*STAR Singapore Institute for Clinical Sciences, Singapore, Singapore

**Keywords:** autoimmune disease, eQTL, comorbidity, protein interaction network, Mendelian randomization, genome-wide association study, juvenile idiopathic arthritis

## Abstract

**Background:**

Juvenile idiopathic arthritis (JIA) is an autoimmune joint disease that frequently co-occurs with other complex phenotypes, including cancers and other autoimmune diseases. Despite the identification of numerous risk variants through genome-wide association studies (GWAS), the affected genes, their connection to JIA pathogenesis, and their role in the development of associated traits remain unclear. This study aims to address these gaps by elucidating the gene-regulatory mechanisms underlying JIA pathogenesis and exploring its potential role in the emergence of associated traits.

**Methods:**

A two-sample Mendelian Randomization (MR) analysis was conducted to identify blood-expressed genes causally linked to JIA. A curated protein interaction network was subsequently used to identify sets of single-nucleotide polymorphisms (i.e., spatial eQTL SNPs) that regulate the expression of JIA causal genes and their protein interaction partners. These SNPs were cross-referenced against the GWAS catalog to identify statistically enriched traits associated with JIA.

**Results:**

The two-sample MR analysis identified 52 genes whose expression changes in the blood are putatively causal for JIA. These genes (e.g., *HLA*, *LTA*, *LTB*, *IL6ST*) participate in a range of immune-related pathways (e.g., antigen presentation, cytokine signalling) and demonstrate cell type-specific regulatory patterns across different immune cell types (e.g., *PPP1R11* in CD4^+^ T cells). The spatial eQTLs that regulate JIA causal genes and their interaction partners were statistically enriched for GWAS SNPs linked with 95 other traits, including both known and novel JIA-associated traits. This integrative analysis identified genes whose dysregulation may explain the links between JIA and associated traits, such as autoimmune/inflammatory diseases (genes at 6p22.1 locus), Hodgkin lymphoma (genes at 6p21.3 [*FKBPL, PBX2, AGER*]), and chronic lymphocytic leukemia (*BAK1*).

**Conclusion:**

Our approach provides a significant advance in understanding the genetic architecture of JIA and associated traits. The results suggest that the burden of associated traits may differ among JIA patients, influenced by their combined genetic risk across different clusters of traits. Future experimental validation of the identified connections could pave the way for refined patient stratification, the discovery of new biomarkers, and shared therapeutic targets.

## Introduction

Juvenile Idiopathic Arthritis (JIA) is an autoimmune disease characterized by chronic joint pain and inflammation (Prakken et al., 2011). Despite being known as a pediatric autoimmune disease, up to 63% of JIA patients maintain active disease into adulthood (Zak and Pedersen, 2000). The exact causes of JIA remain unknown. However, it has high heritability (Li et al., 2015) and an increased concordance rate in monozygotic twins (Savolainen et al., 2000), strongly suggesting genetic contributions to disease pathogenesis.

The majority of the genetic loci associated with JIA are located in the non-coding regions of the genome (Thompson et al., 2012; Hinks et al., 2013; López-Isac et al., 2021). Studies have demonstrated that disease-associated variants (e.g., Single nucleotide polymorphisms [SNPs]) are enriched within regulatory DNA elements (Nicolae et al., 2010; Ernst et al., 2011; Maurano et al., 2012). One of the key mechanisms of gene regulation involves direct physical interaction between distal regulatory elements and their target genes (Schoenfelder et al., 2010; Panigrahi and O'Malley, 2021). Previous work by our group has investigated how JIA-associated SNPs influence the expression of distant genes through SNP-gene physical interaction (Pudjihartono et al., 2023). While these findings provided valuable insights, they fell short of providing statistical evidence for the causal involvement of these genes in JIA. Recently, Mendelian randomization (MR) has gained traction as an effective strategy for identifying genes that have causal roles in disease pathogenesis (Davey Smith and Hemani, 2014; Hartwig et al., 2016). We contend that by using variants that physically interact and are associated with gene expression (termed “spatial eQTL SNPs”) as instrumental variables and GWAS data to assess its impact on disease outcome, we can utilize MR to infer a causal relationship between altered gene expression and specific disease outcomes (Baiocchi et al., 2014).

Comorbidity between JIA and other conditions, such as type 1 diabetes (Szabłowski et al., 2022), inflammatory bowel disease (van Dijken et al., 2011; Barthel et al., 2015), and cancers (Nordstrom et al., 2012; Horne et al., 2019a) have been widely reported. This suggests the existence of shared molecular pathways between these traits (Nagy et al., 2018; Zolotareva et al., 2019). Understanding the biological underpinnings of JIA and how they contribute to the intersection with these associated conditions could illuminate the mechanisms behind comorbid disease development and aid in identifying shared therapeutic targets. However, the gene-regulatory mechanisms linking JIA to many of its associated (both positively and negatively associated) traits remain elusive.

In this study, we performed two-sample MR using spatial eQTLs within a blood gene regulatory network (GRN) as instrumental variables to identify 52 potential JIA causal genes. These genes are involved in immune response-related pathways and exhibit interesting patterns of gene regulation specific to various immune cell types. We hypothesized that the association between different traits could occur due to the dysregulation of common biological pathways. Therefore, we identified sets of spatial eQTLs within the blood GRN that regulate JIA causal genes or its protein interaction partners. These SNPs were statistically enriched for 95 GWAS traits, covering a range of autoimmune and inflammatory diseases, cancers, and immune-related protein levels. Many of these traits have previously been found to be positively associated with JIA (e.g., type 1 diabetes, blood cancers, C-reactive protein levels), while others (e.g., sphingomyelin levels, platelet-to-lymphocyte ratio, multiple sclerosis) represent potentially novel associations. Notably, we identified gene clusters that might mediate the association between JIA and different groups of traits (e.g., 6p22.1 linked to certain autoimmune/inflammatory diseases, 6p21.3 linked to Hodgkin lymphoma). Collectively, our results provide new insights into the biological mechanisms behind JIA and its potential role in the emergence of associated traits.

## Materials and methods

### Creation of the blood GRN

The blood GRN was constructed by identifying spatial eQTLs involving all common SNPs (MAF ≥ 0.05; n = ∼40 × 10^6^) present within the whole blood eQTL dataset (from Genotype-Tissue Expression Project [GTEx V8]) (Aguet et al., 2020). In this study, spatial eQTLs are defined as SNPs that tag a locus, which regulates the expression of target genes via physical interaction. This was accomplished using the CoDeS3D pipeline (https://github.com/Genome3d/codes3d-v2) (Fadason et al., 2017). In brief, high-resolution Hi-C chromatin contact data from four primary blood cell lines (Rao et al., 2014) (Supplementary Table S1) was used to identify target genes that interact with restriction fragments containing the input SNPs. Only SNP-gene interactions captured in ≥1 Hi-C cell lines were included in subsequent eQTL analysis. eQTL data from GTEx whole blood samples (Aguet et al., 2020) was queried to identify spatial eQTLs. Multiple testing correction was done using the Benjamini-Hochberg procedure, where spatial eQTL – target gene pairs with an adjusted *p*-value ≤ 0.05 were selected as significant.

### Two-sample Mendelian randomization

To identify potentially causal genes for JIA within the blood GRN, we conducted a two-sample Mendelian randomization using the TwoSampleMR R package (https://github.com/MRCIEU/TwoSampleMR/, version 0.5.6) (Hemani et al., 2018). This analysis adhered to the STROBE-MR guidelines (Supplementary Material) (Skrivankova et al., 2021). MR relies on three main assumptions. First, the genetic instruments (i.e., spatial eQTLs) must be robustly associated with the exposure of interest (i.e., gene expression). Second, these instruments should be independent of any potential confounders. Third, the genetic instruments influence the outcomes solely through their association with the exposure (i.e., no horizontal pleiotropy). To satisfy the first assumption, we used only statistically significant spatial eQTL-gene pairs within the blood GRN (adjusted *p*-value ≤0.05) as exposure instruments. Furthermore, to ensure that instrumental variables for each exposure were independent, we performed linkage disequilibrium (LD) clumping with r^2^ cutoff of 0.001. For this, the European (EUR) population from the 1000 Genomes project (Auton et al., 2015) served as the reference panel for LD analysis. A recent JIA GWAS by López-Isac et al. (López-Isac et al., 2021), comprising 3,305 JIA cases and 9,196 controls, was selected for the outcome data (https://www.ebi.ac.uk/gwas/downloads/summary-statistics) (Study Accession Code: GCST90010715). After harmonizing the exposure and outcome data, genes with one instrumental variable underwent 2SMR using the Wald test, whereas those with two instrumental variables underwent two-sample MR using the inverse variance weighted (IVW) method, those with 3 or more instrumental variables underwent two-sample MR using IVW and weighted median methods. Genes whose MR *p*-value was equal to or below the Bonferroni-corrected threshold (0.05/number of unique exposure genes [13,640]) were considered statistically significant (Supplementary Table S2). For genes with ≥2 instrumental variables, Cochran’s Q was computed to quantify the variation in causal effect estimates attributed to different instruments. A *p*-value ≤ 0.05 suggests significant heterogeneity, which may indicate pleiotropy or other issues such as invalid instrumental variables. For genes with ≥3 instrumental variables we also performed an MR-Egger regression to test for horizontal pleiotropy by evaluating its intercept. A significant non-zero intercept (*p*-value ≤ 0.05) is considered evidence of horizontal pleiotropy. Finally, exposure genes that failed to pass these sensitivity analyses were removed from the final causal gene list (Supplementary Table S3).

### Genes previously associated with JIA

Genes previously linked with JIA were identified by referencing the GWAS catalog (Supplementary Table S4, column = Mapped Gene) and querying DisGeNET, a gene-disease association public repository (Pinero et al., 2015). From DisGeNET, the “Curated gene-disease associations” file (https://www.disgenet.org/; April 22^nd^, 2022) was downloaded. Genes linked to JIA were isolated by extracting entries corresponding to the disease names “Juvenile arthritis” or “Juvenile Pauciarticular chronic arthritis” (Supplementary Table S5).

### Gene Ontology and KEGG pathway enrichment analysis

We employed g:profiler (Raudvere et al., 2019) (accessed on May 17th, 2022, from https://biit.cs.ut.ee/gprofiler/gost) to discern significantly enriched biological processes and pathway terms among JIA causal genes. Our analysis encompassed both Gene Ontology (specifically, the biological process category) and the Kyoto Encyclopedia of Genes and Genomes (KEGG pathway). The Benjamini-Hochberg procedure was utilized for multiple testing corrections, and terms with an adjusted *p*-value of 0.05 or less were considered significant (Supplementary Tables S6, S7).

### Immune cell type-specific analysis of causal gene regulation

We assessed the eQTL effect of the instrumental variable (IV) SNPs for each of the 52 causal genes across 15 immune cell types, utilizing the Database of Immune Cell eQTLs (DICE). This analysis was accomplished using the CoDeS3D pipeline. Multiple testing correction was done using the Benjamini-Hochberg procedure, where eQTL–target gene pairs with an adjusted *p*-value ≤ 0.05 in each immune cell type were selected as significant (Supplementary Table S8).

Hierarchical clustering of the effect size (beta) of eQTL-target gene pairs was performed using the pheatmap R package (version 1.0.12). It’s noteworthy that we limited our clustering analysis to the same gene relationships identified in the two-sample MR analysis. For instance, since SNP rs165256 served as the instrumental variable for the *PPP1R11* gene in our two-sample MR analysis, we exclusively considered the eQTL effect of rs165256 with the *PPP1R11* gene only. Here, the magnitude direction of the effect size for the eQTL – target gene pair was determined by the risk allele of the eQTL SNP. However, by default, CoDeS3D reports the effect size magnitude based on the alternate allele of each SNP in dbSNP151 — a public database detailing genetic variations aligned with the forward/+ strand of the GRCh38 reference genome. However, the alternate allele might not always correspond to the risk allele. To determine the risk allele of an SNP, we queried the “harmonized data” data frame outputted by the “harmonise_data()” function in the TwoSampleMR R package (Supplementary Table S9). Here, if the “beta outcome” column shows a positive number, the alternate allele of the related SNP is a risk factor for JIA. Conversely, a negative number implies the risk allele is the SNP’s reference allele. In instances where the risk allele of the eQTL SNP matches its reference allele (and not the alternate allele), we adjusted the effect size direction provided by CoDeS3D (i.e., switching −1 to +1 or *vice versa*). This adjustment ensures the gene regulatory direction (either up- or downregulation) aligns accurately with the risk allele of each SNP.

### Identification of traits sharing biological interactions with JIA

Potentially associated (both positively or negatively) traits were identified using a version of the Multimorbid3D (Pudjihartono et al., 2024) pipeline (https://github.com/MichaelPudjihartono/multimorbid3D), with the 52 JIA causal genes as the input. Briefly, the STRING database (v11.5) (Szklarczyk et al., 2021) was queried to create a protein-protein interaction (PPI) network (Supplementary Table S10), encompassing both physical interactions and functional associations. The interaction data were obtained using the following criteria: experiments, text-mining of the scientific literature, previous knowledge in databases, co-expression, neighborhood, gene fusion, and co-occurrence, species limited to “*Homo sapiens*”, and interaction score > 0.9.

The resultant network extended beyond the initial 52 causal genes, spanning two additional levels (levels 0–2). The “level 0”network comprised the proteins encoded by the 52 causal genes, while each successive level encompassed proteins interacting with those from the previous level (Supplementary Table S11). At each level, the genes encoding the proteins were queried to the blood GRN to identify spatial eQTLs regulating the expression of the genes. The set of spatial eQTLs at each level were then queried to the GWAS catalog to identify traits whose GWAS SNPs overlap with the identified spatial eQTLs (or its LD partners within r^2^ ≥ 0.8). This consideration of LD partners of a spatial eQTL is to account for the fact that GWAS-identified SNPs usually represent an entire genetic locus (Pudjihartono et al., 2022). Then, at each level, statistically significant GWAS trait enrichments were determined by hypergeometric test (*P* ≤ 0.05). The Benjamini-Hochberg procedure was used to adjust the *p*-values (FDR ≤ 0.05). Additionally, bootstrapping (n = 240) was performed by randomizing the input gene set equal to the size of the original input gene (i.e., 52 genes). For each trait, the bootstrapping *p-*value was determined using the formula:

$$p=\frac{\text{number}\;\text{of}\;\text{simulations}\;\text{for}\;\text{which}\;\text{the}\;\text{trait}\;\text{in}\;\text{question}\;\text{is}\;\text{significant}+1}{240+1}$$

Traits with hypergeometric adjusted *p-*value and bootstrapping *p-*value ≤ 0.05 were deemed to be significantly associated with JIA (Supplementary Table S12, S13). It should be noted that because there is no constraint on the GWAS SNP direction of effect, the identified traits could be positively or negatively associated with JIA. To visualize the contributions of different genes to various traits across each level of the PPIN, hierarchical clustering on the log number of GWAS SNP of each significant trait that overlaps with spatial eQTLs (or its LD partners; Supplementary Table S14) targeting gene at different PPIN levels was performed using the pheatmap R package (version 1.0.12).

## Results

### Creation of the spatially constrained blood gene regulatory networks (GRN)

**FIGURE 1:**
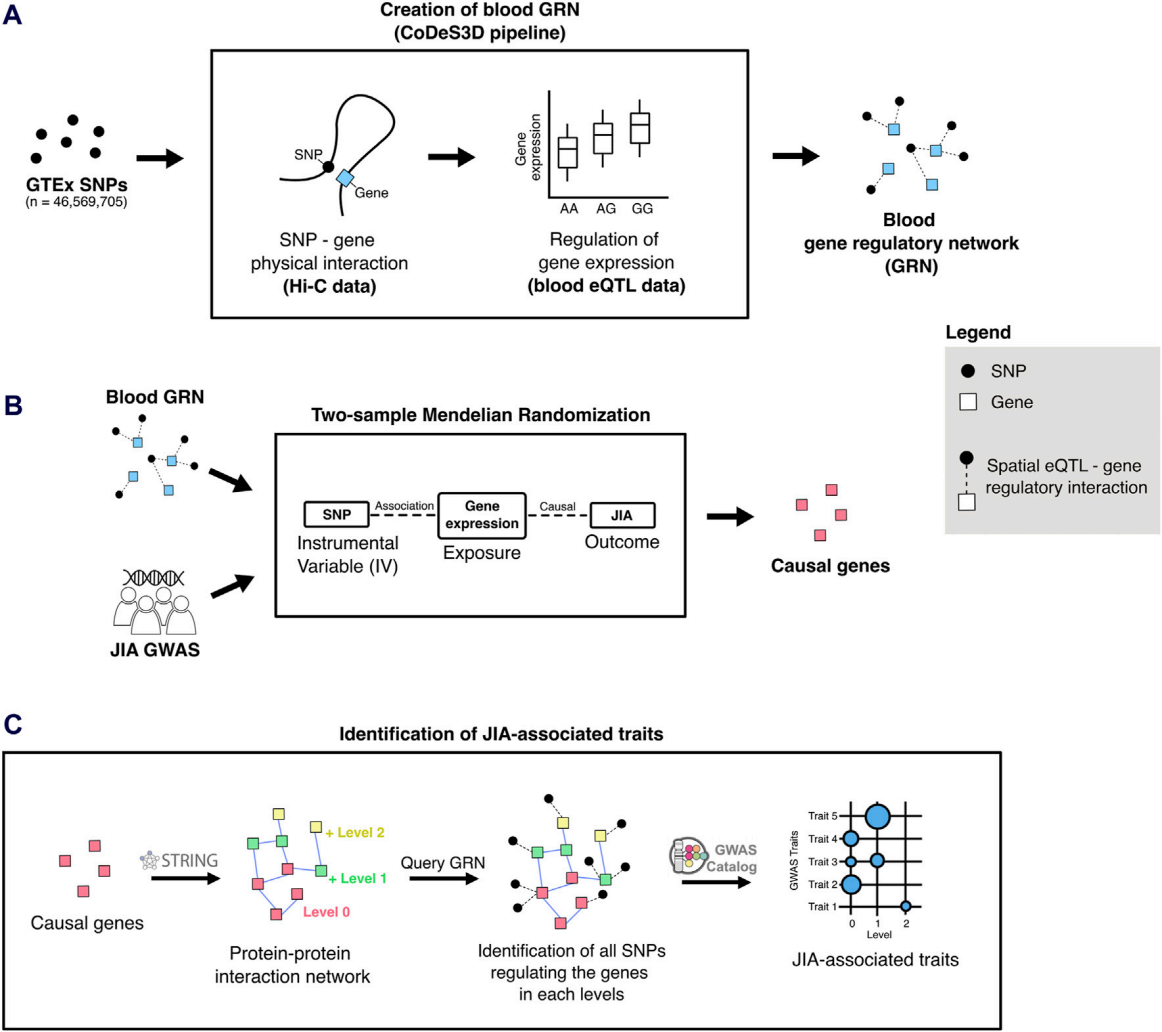
Overview of the analytical approach used in this study. **(A)** The blood-specific gene regulatory network (GRN) was generated using the CoDeS3D pipeline (Fadason et al., 2017). **(B)** Two-sample Mendelian randomization uses SNPs associated with gene expression changes (i.e., spatial eQTLs from the blood GRN) to infer causal relationships between gene expression and JIA outcomes. **(C)** To identify JIA-associated traits, the STRING (Szklarczyk et al., 2021) database was queried to identify proteins that interact up to two edges away (levels 1 and 2) from the JIA causal genes (level 0). The blood GRN was queried to identify the sets of spatial eQTLs that regulate the expression the genes on each level. Finally, these sets of spatial eQTLs and their linkage disequilibrium (LD) partners (r^2^ ≥ 0.8) were tested for overlap and enrichment for SNPs associated with different traits in the GWAS Catalog.

The CoDeS3D pipeline (Fadason et al., 2017) was used to analyse common SNPs in the human genome (Minor Allele Frequency ≥ 0.05) to identify spatial eQTLs (i.e., SNPs that tag a locus that physically interacts with a gene and associates with its expression levels) within the GTEx whole blood eQTL dataset (Aguet et al., 2020) (Figure 1A). We used Hi-C physical contact data from four primary blood cell lines (Rao et al., 2014) (Supplementary Table S1) in the construction of the GRN. The resulting blood GRN comprised 1,713,885 spatial eQTL – target gene interactions (1,077,379 SNPs and 14,871 target genes expressed in whole blood (Zaied et al., 2023).

### Two-sample MR identified 52 potential JIA causal genes enriched within immune-related pathways in the blood GRN

**FIGURE 2:**
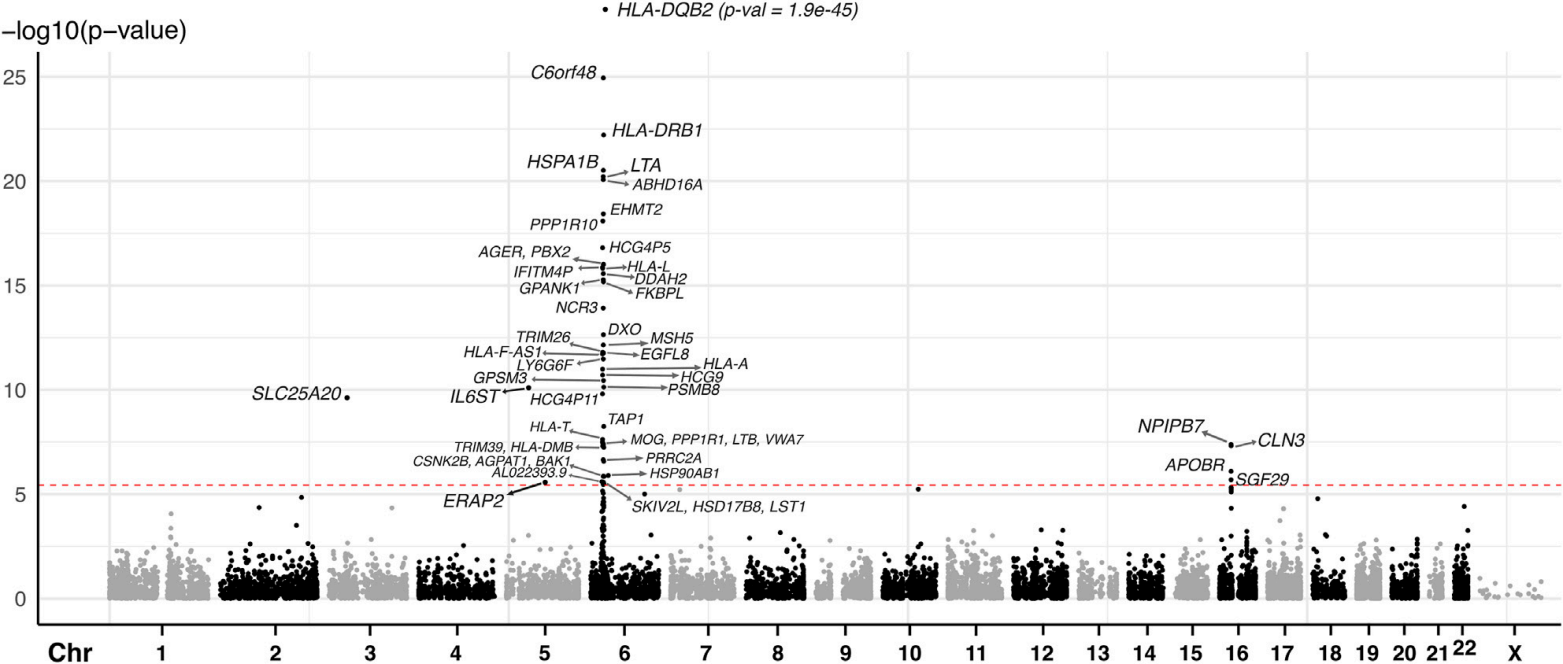
Manhattan plot of JIA causal genes identified using spatial eQTLs within the blood GRN and JIA GWAS. The red dashed line indicates the Bonferroni-corrected MR causal effect *p-*value threshold (*p =* 3.607 × 10^−6^). Each dot represents the starting coordinate of a gene.

We conducted a two-sample MR analysis using spatial eQTLs from the blood GRN as genetic instrumental variables and JIA GWAS summary statistics (3,305 cases and 9,196 healthy controls) (López-Isac et al., 2021) as the outcome data (Figure 1B). This analysis was conducted in accordance with the STROBE-MR guidelines (Skrivankova et al., 2021) (Supplementary Material). Our analysis identified 52 genes whose expression changes in the blood were putatively causal for JIA (hereafter referred to as “causal genes”; Table 1; Supplementary Table S2). Most of the identified genes (n = 44; 85%) were located within the HLA locus on chromosome 6 (i.e., 6p21.3 – 6p22.1; Figure 2; Supplementary Table S2), which is the most variable region in the genome and known for its immune-regulatory functions (Shiina et al., 2009). While previous GWAS findings have shown that variants within this locus are associated with JIA (Hinks et al., 2013; López-Isac et al., 2021), our study provides evidence for the causal impact of altered gene expression within this locus. Notably, the vast majority (n = 51; 98%) of the identified causal genes have not been previously linked to JIA, either in DisGeNET (Pinero et al., 2015) (Supplementary Table S5) or through the “nearest genes” approach represented in the GWAS catalog (Supplementary Table S4; Supplementary Figure S1).

**TABLE 1:** Two-sample MR results using spatial eQTLs within the blood GRN and JIA GWA**S**. The odds ratio shows the multiplicative effects of each unit increase in gene expression on the odds of the disease. Therefore, an OR > 1(red) signifies that increased expression of a gene is associated with elevated JIA risk, whereas an OR < 1 (blue) denotes the opposite effect. The full table is in Supplementary Table S2.

Gene	Instrumental variables	Method	Odds ratio	MR *p-*value
HLA-DQB2	rs2621331	Wald ratio	**9.60**	1.90E-45
HLA-DRB1	rs9469220	Wald ratio	**0.02**	6.13E-23
LTA	rs3219190	Wald ratio	**10.81**	6.09E-21
HCG4P5	rs2517714	Wald ratio	**1.89**	1.56E-17
AGER	rs204995	Wald ratio	**0.15**	9.80E-17
PBX2	rs204995	Wald ratio	**7.94**	9.80E-17
IFITM4P	rs9368609	Wald ratio	**1.48**	1.37E-16
FKBPL	rs12153855	Wald ratio	**0.31**	6.71E-16
MSH5	rs2395153, rs7764682	Inverse variance weighted	**0.06**	7.15E-13
TRIM26	rs929156	Wald ratio	**0.24**	1.56E-12
HLA-A	rs9260114	Wald ratio	**3.88**	1.01E-11
HCG9	rs1610586	Wald ratio	**1.82**	1.94E-11
GPSM3	rs28752784	Wald ratio	**0.004**	3.55E-11
IL6ST	rs13186299	Wald ratio	**0.17**	7.96E-11
TAP1	rs3763348	Wald ratio	**4.40**	5.64E-09
HLA-T	rs9265961	Wald ratio	**1.43**	2.35E-08
MOG	rs1611284	Wald ratio	**0.42**	3.12E-08
PPP1R11	rs165256	Wald ratio	**0.16**	3.44E-08
LTB	rs2239704	Wald ratio	**0.08**	3.81E-08
TRIM39	rs2516719	Wald ratio	**0.17**	4.98E-08
PRRC2A	rs2844472	Wald ratio	**3.49**	2.22E-07
HSP90AB1	rs10807029	Wald ratio	**13.82**	1.27E-06
AGPAT1	rs3134950	Wald ratio	**0.05**	1.40E-06
BAK1	rs210142	Wald ratio	**0.78**	1.41E-06

Gene enrichment analysis [g:Profiler (Raudvere et al., 2019)] of the causal genes revealed enrichment in 51 biological processes (Gene Ontology) and KEGG pathway terms (FDR < 0.05; Supplementary Table S6). These terms are pre-dominantly immune system-related processes, pathways, or diseases (e.g., antigen processing and presentation; immune system process; immune cell activation and proliferation; cytokine signalling; type 1 diabetes; autoimmune thyroid disease). Notably, all 51 significantly enriched terms included HLA class I or II genes, consistent with recognized roles for HLA genes in the immune system (Choo, 2007). To explore the functional impact of non-HLA genes, we excluded the 7 HLA class I or class II genes from the gene set and repeated the enrichment analysis. We identified three immune-related Gene Ontology terms that exhibited significant enrichment; two of them are related to the regulation of cytokine signaling (Supplementary Table S7; 9 genes; *GPSM3, AGPAT1, LTB, AGER, IL6ST, PPP1R11, HSP90AB1, MOG, LTA*).

### Cell type-specific eQTL data reveals distinct patterns of causal gene regulation across 15 immune cell types

**FIGURE 3:**
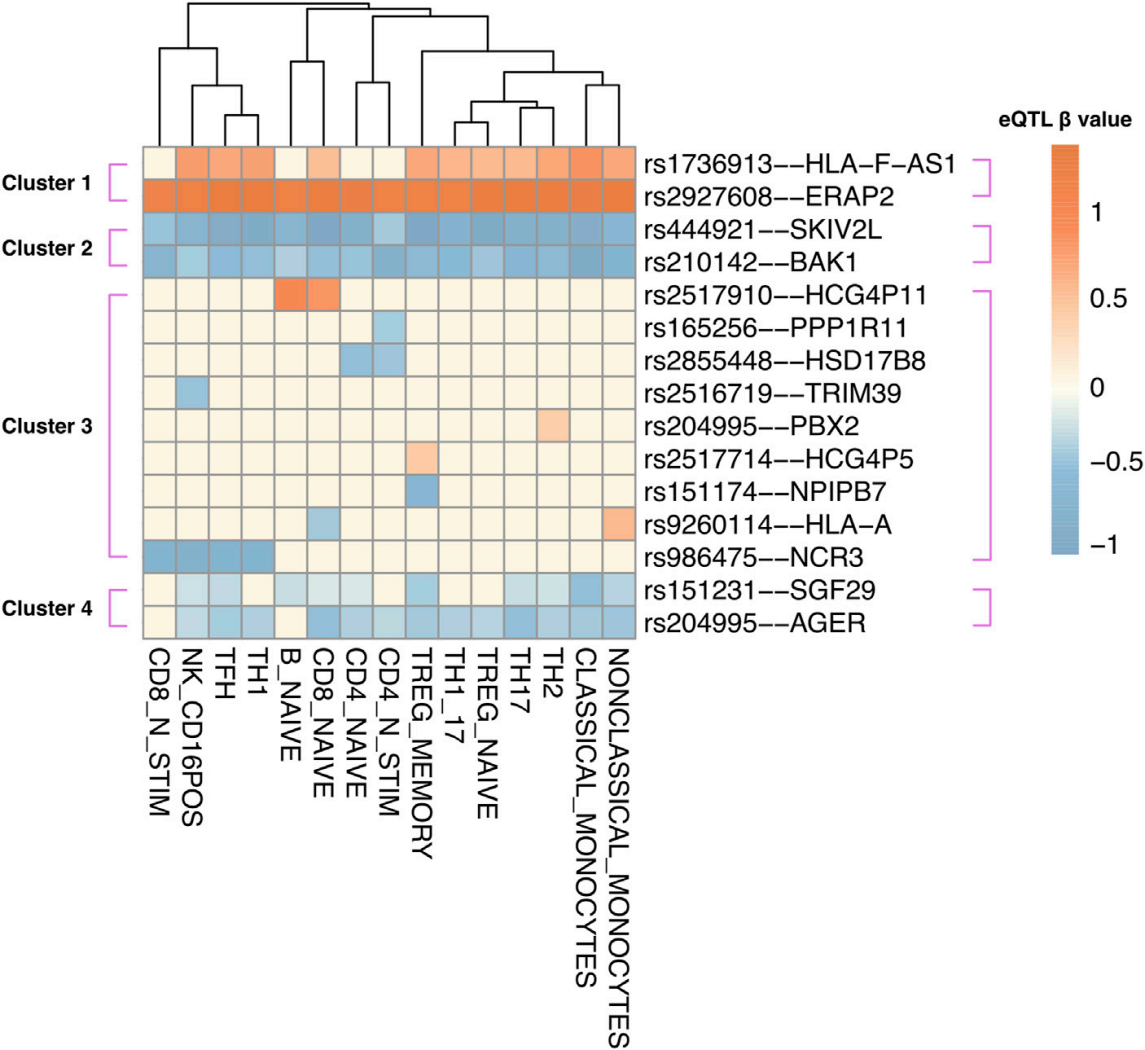
Instrumental variable (IV) SNPs exhibit immune cell type-specific causal gene regulatory effects. eQTL – target gene pairs in 15 DICE immune cell types were hierarchically clustered according to eQTL beta coefficient. The beta coefficient (red: upregulation; blue: downregulation) was shown according to the risk allele of each instrumental variable SNP.

Human blood is a heterogeneous tissue consisting of specialized cells that include different immune cell lineages. To investigate the cell type-specific regulatory patterns of the 52 causal genes that were identified in whole blood, we tested the instrument SNPs of each causal gene for eQTL effects using the Database of Immune Cell eQTLs (DICE (Schmiedel et al., 2018); Supplementary Table S8). This was accomplished using the CoDeS3D pipeline. Of the 52 causal genes, the transcript levels of 15 genes were associated with the same SNPs within the DICE dataset, as they were in two-sample MR. Hierarchical clustering of these eQTL–target gene pairs identified four visually distinct clusters (Figure 3). Cluster 1 includes target genes that are upregulated by eQTLs across most immune cell types, whereas clusters 2 and 4 consist of genes that are downregulated in most immune cell types. For instance, the *ERAP2* transcript levels are upregulated by rs2927608, while the *BAK1* transcript levels are downregulated by rs210142 across 15 immune cell types. By contrast, cluster 3 consists of target genes that exhibit cell-type specific regulatory patterns. For instance, the instrumental variable rs165256 was associated with the downregulation of the *PPP1R11* gene specifically in activated CD4^+^ T helper cells. We contend that understanding the functional role of these cell type-specific genes is crucial for comprehending how different immune cell types contribute to the etiology of JIA.

### Spatial eQTLs regulating JIA causal genes and their protein interaction partners show enrichment for JIA-associated traits

**FIGURE 4:**
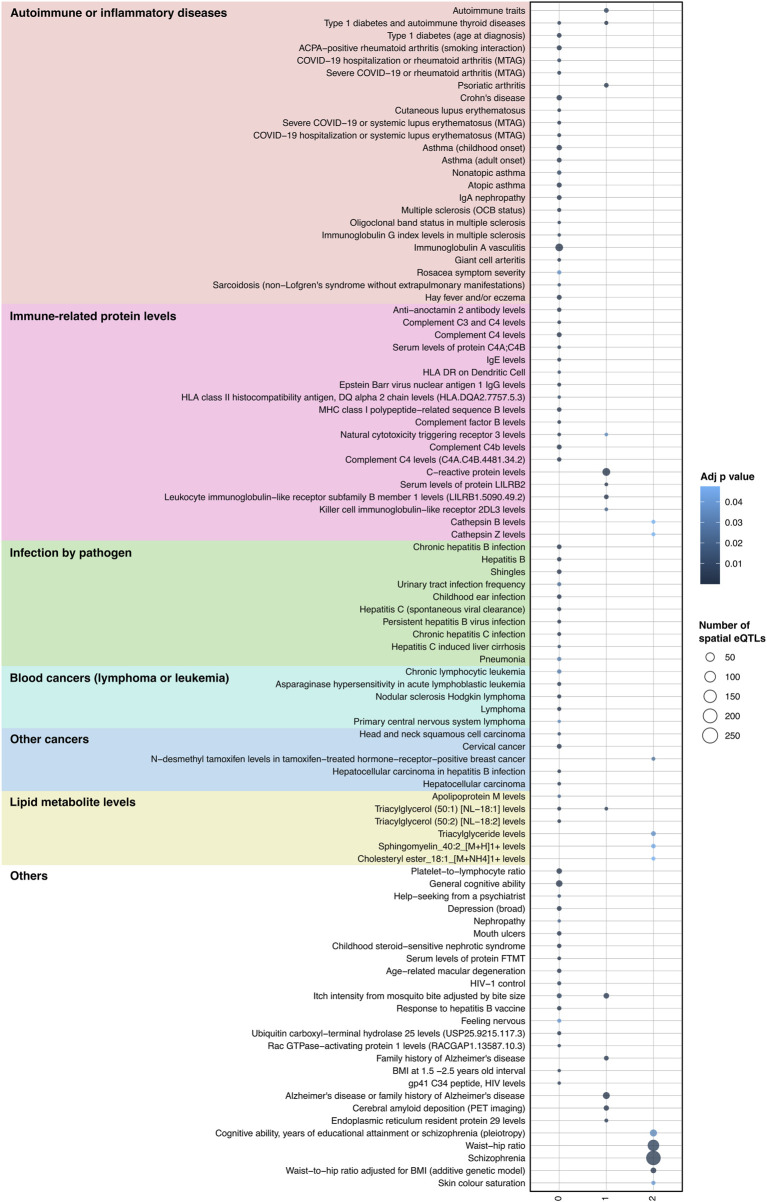
Integrating protein-protein interactions and GRN with GWAS data identifies traits associated with JIA. A total of 95 traits were identified as enriched (hypergeometric tests, FDR ≤ 0.05, and bootstrapping n = 240) within the spatial eQTLs that target JIA causal genes (level 0), genes directly interacting with causal genes (level 1) and genes interacting with level 1 genes (level 2). Physiologically similar traits are color coded and grouped. The X-axis represents the different PPIN levels.

We posited that a subset of JIA causal genes exhibit pleiotropy (i.e., a single gene influences two or more phenotypic traits). Given that proteins operate interdependently, it was conceivable that these genes, along with their protein interaction partners, might form the biological link connecting JIA with other complex traits. Therefore, the STRING database (Szklarczyk et al., 2021) was used to create a protein-protein interaction (PPI) network centered on the causal genes (Figure 1C). This PPI network extends beyond the initially identified JIA causal genes by two levels, where each level contains proteins that interact with proteins encoded by genes from the preceding level (Supplementary Table S10). The resulting PPI network consisted of 52 proteins in level 0, 495 proteins in level 1, and 3,846 proteins in level 2 (Supplementary Table S11). Subsequently, the blood GRN was queried to identify the set of spatial eQTLs that regulate the expression of the genes on each level. These sets of spatial eQTLs and their LD partners (r^2^ ≥ 0.8) were tested for overlap and enrichment for SNPs associated with traits in the GWAS Catalog using a hypergeometric test (adjusted *p-*value and bootstrapping *p-*value ≤ 0.05). Across the three PPIN levels (level 0–2), we identified enrichment for 95 GWAS traits (Figure 4; Supplementary Table S12). The largest category of associated traits was “autoimmune or inflammatory diseases” (22 traits at level 0; 3 traits at level 1) and included conditions such as rheumatoid arthritis, psoriatic arthritis, eczema, and asthma. This was followed by “immune-related protein levels” (13 traits at level 0; 5 traits at level 1; 2 traits at level 2; e.g., complement system protein levels, IgE levels). We also observed traits associated with infection (10 traits at level 0; e.g., hepatitis B/C, shingles), cancers (9 traits at level 0; 1 trait at level 2; e.g., lymphomas, leukemias, carcinomas), and lipid metabolite levels (3 traits at level 0 and 2; 1 trait at level 1). Interestingly, our analysis identified numerous traits and diseases with established links to JIA (refer to Table 2), as well as others whose connections to JIA remain to be validated (e.g., Sphingomyelin levels, IgA nephropathy, giant cell arteritis).

**TABLE 2:** Identified traits that have known positive associations with JIA. Here, we consider evidence of comorbidity and correlations with disease severity.

Trait	Level (0–2)	References(s)
Type 1 diabetes	0	Hermann et al. (2015), Szabłowski et al. (2022)
Crohn’s disease	0	van DIJKEN et al. (2011), Barthel et al. (2015)
Asthma	0 and 1	Lin et al. (2016)
Psoriatic arthritis	1	Brunello et al. (2022)
Atopic dermatitis	0	Keskitalo et al. (2023)
Multiple sclerosis*	0	Tseng et al. (2016)
Complement C4 levels	0	Jarvis et al. (1993), Gilliam et al. (2011)
Complement factor B levels	0	Jarvis et al. (1993)
Cathepsin B levels*	2	Ikeda et al. (2000), Behl et al. (2022)
C-reactive protein levels	1	Gwyther et al. (1982), Gilliam et al. (2008), Swart et al. (2016)
Platelet-to-lymphocyte ratio*	0	Milovanovic et al. (2004), Zha et al. (2006), Fu et al. (2015), Gasparyan et al. (2019)
Epstein Barr virus infection/plasma antibody level	0	Opoka-Winiarska et al. (2020)
LILRB2 protein level*	1	Huynh et al. (2007)
IgE levels	0	Poddighe et al. (2021)
Shingles	0	Nimmrich and Horneff (2015)
Pneumonia	0	Beukelman et al. (2012)
Hepatitis B*	0	Chen et al. (2018)
General cognitive ability	0	Mena-Vázquez et al. (2022)
Lymphoma and leukemia	0	Marwaha et al. (2010), Zombori et al. (2013), Horne et al. (2019a)
Tonsillectomy	1	Astrauskiene et al. (2009)
Sphingomyelin levels*	2	Beckmann et al. (2017)

Traits marked with an asterisk (*) are known to be associated with adult arthritis but have not been definitively linked to JIA.

### Shared dysregulation of pleiotropic JIA causal genes connects JIA to associated traits

**FIGURE 5:**
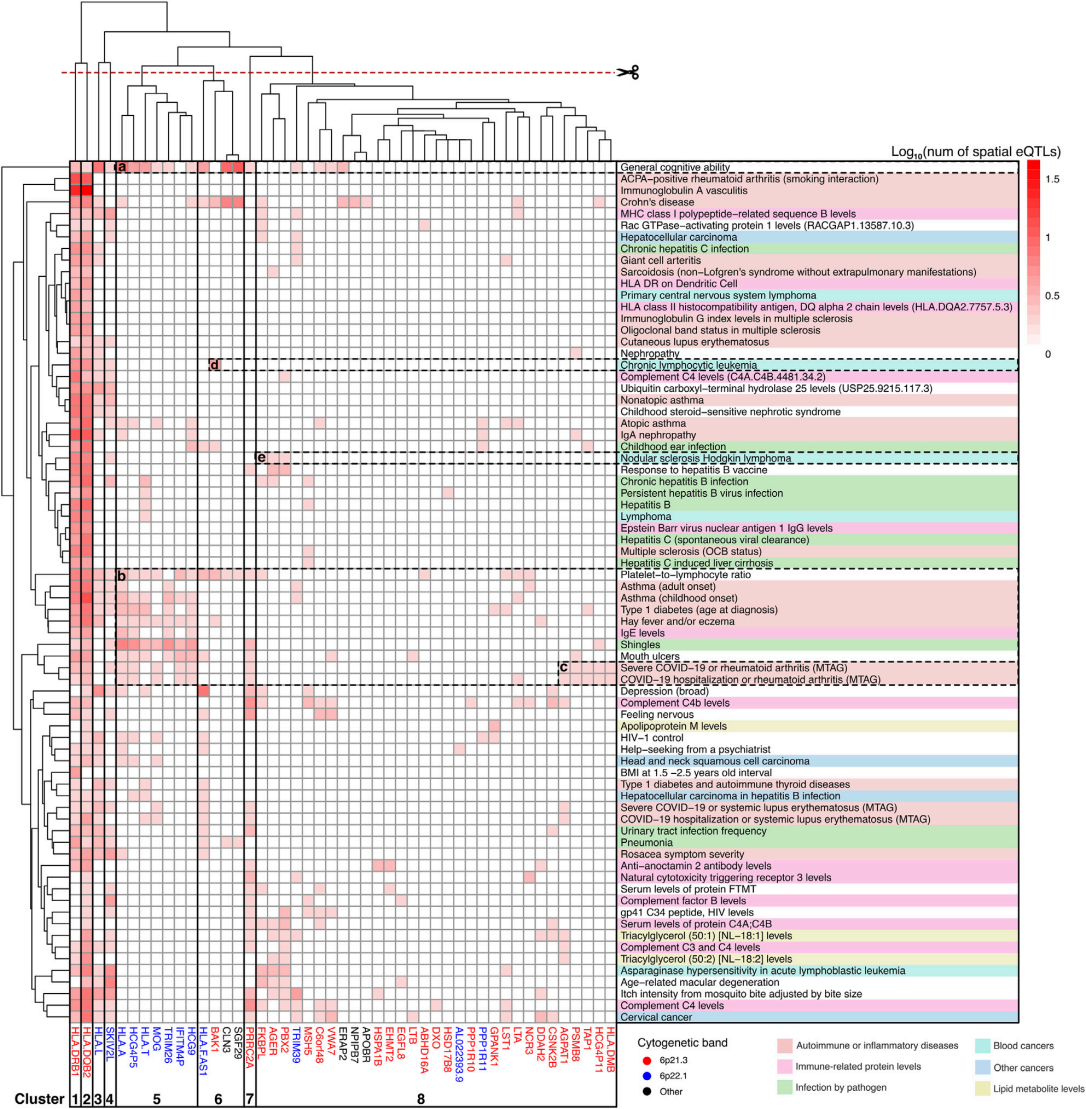
Clusters of JIA causal genes drive associations with level 0 traits. Gene-trait associations was hierarchically clustered according to the number of GWAS SNPs that overlapped (or were in LD r^2^ ≥ 0.8 with) spatial eQTLs regulating different level 0 genes. Genes are color-coded according to their chromosomal location. Traits are categorized and color-coded as in Figure 3. See Supplementary Table S14 for raw data.

Hierarchical clustering was used to organize gene-trait associations at different levels of the PPIN (Supplementary Figure S2). This analysis yielded insights into the contributions of various genes to different groups of associated traits. For example, at level 0 (see Figure 5), clusters 1 and 2 comprised two highly pleiotropic HLA class II genes whose expression levels were linked to spatial eQTLs that were GWAS-associated with most level 0 traits. Cluster 5, which includes genes at 6p22.1 (*HLA-A, HCG4P5, HLA-T, MOG, TRIM26, HCG9, IFITM4P*), demonstrated further connections between JIA and a subset of autoimmune/inflammatory traits (i.e., type 1 diabetes, asthma, eczema, and severe COVID-19 or rheumatoid arthritis). Additionally, these cluster 5 genes also contributed to other traits including the platelet-to-lymphocyte ratio, IgE level, general cognitive ability, and shingles (see Figures 5a,b). Some of these traits (i.e., platelet-to-lymphocyte ratio, general cognitive ability) as well as Crohn’s disease are further linked to JIA through the neighbouring cluster 6 genes (*HLA-FAS1, BAK1, CLN3, SGF29*). We observed the distinct association between “severe COVID-19 or rheumatoid arthritis” with JIA that was mediated by a subset of 6p21.3 genes within cluster 8 (*AGPAT1, PSMB8, TAP1, HCG4P11, HLA-DMB*; Figure 5c). Among cancer traits, in addition to the highly pleiotropic genes in clusters 1-4, JIA has a unique association with “nodular sclerosis Hodkin lymphoma” through a subset of genes in cluster 8 (*FKBPL, PBX2, AGER*; Figure 5e) and with “chronic lymphocytic leukemia” through the *BAK1* gene in cluster 6 (Figure 5d).

### Protein-protein interactions reveal a novel gene regulatory link between JIA and psoriatic arthritis

**FIGURE 6:**
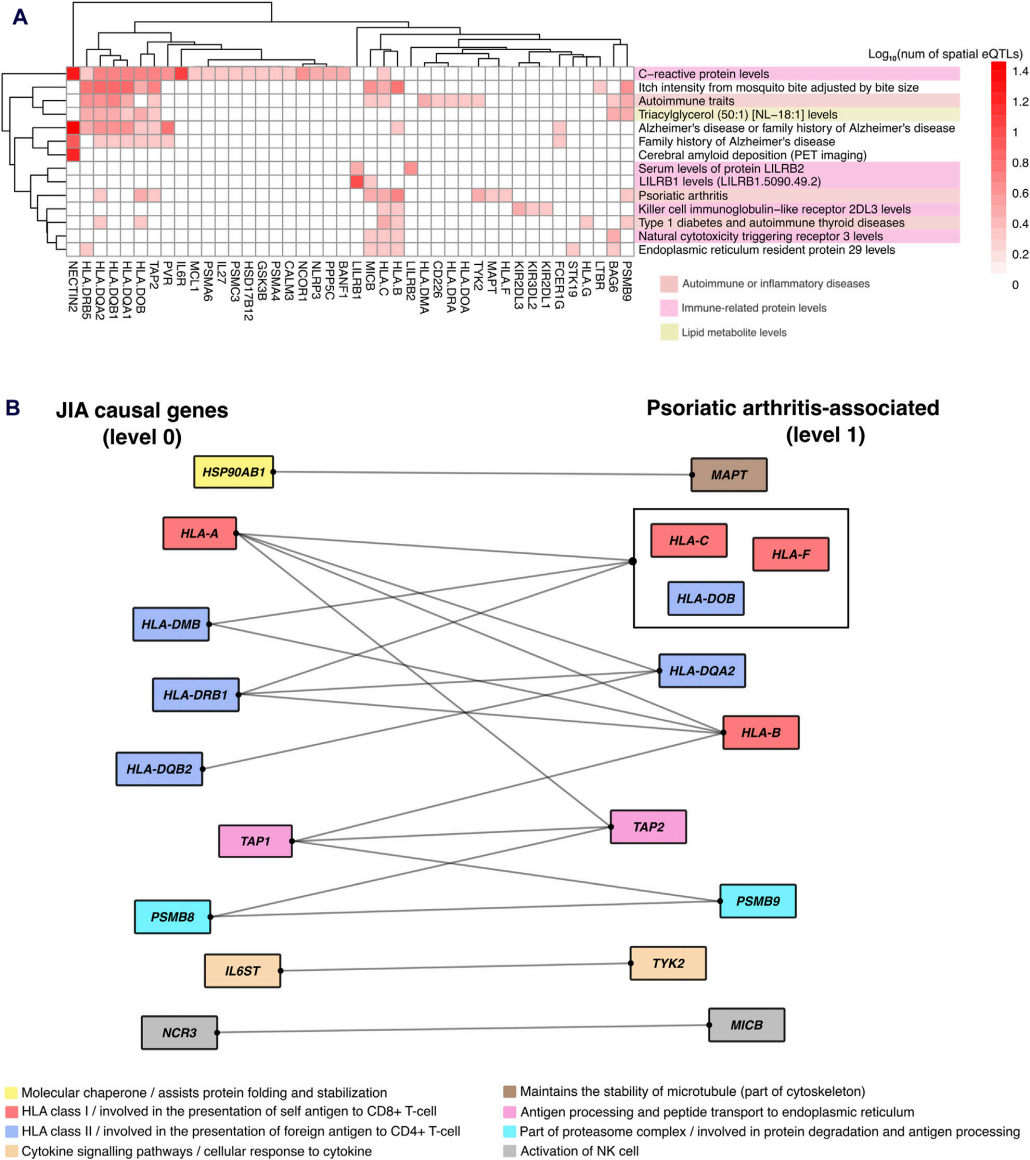
Protein-protein interactions reveal gene regulatory links between JIA and associated traits. **(A)** Hierarchical clustering of gene-trait associations occurring at level 1 of the JIA PPIN. Clustering was based on the number of GWAS SNPs that overlapped (or in LD r^2^ ≥ 0.8 with) spatial eQTLs regulating level 1 genes. **(B)** Protein-protein interaction between JIA causal genes and genes regulated by psoriatic arthritis-associated spatial eQTLs. Protein interactions are represented by edges. Proteins that share the same interactions are grouped together. Proteins are color-coded based on their cellular functions.

The identification of enriched traits for spatial eQTLs at the outer level of the PPIN (i.e., level 1–2) indicated that the convergence between JIA and associated traits occurred indirectly through protein-protein interactions. One notable example is “psoriatic arthritis” (level 1). Juvenile psoriatic arthritis (or JPsA) is one of the 7 subtypes of JIA recognized by the International League of Associations of Rheumatology (ILAR) (Petty et al., 2004). Individuals with Juvenile psoriatic arthritis experience joint inflammation and exhibit extra-articular symptoms associated with psoriasis (e.g., scaly and itchy skin plaques). Our result showed that 10 proteins within level 1 of JIA PPIN that are regulated by spatial eQTLs associated with psoriatic arthritis (Figures 6A, B) interact with proteins encoded by 9 JIA causal genes (level 0; Figure 6B). These protein interactions suggest that dysregulation of a shared pathway potentially explains the link between JIA and psoriasis.

## Discussion

In this study, we conducted a two-sample MR analysis on JIA by integrating large-scale GWAS and spatial eQTL data obtained from whole blood samples. Our analysis identified 52 genes with potential causal roles in JIA, most of which are located within the HLA locus on chromosome 6p21.3–6p22.1. The two-sample MR method considers both GWAS and eQTL *p*-values to assign causality to a gene (Zhu and Zhou, 2021). Therefore, the abundance of causal genes within the HLA locus is consistent with previous GWAS results, which showed the strongest association signals for SNPs within the extended HLA region (Hinks et al., 2013; López-Isac et al., 2021). The novelty of our finding lies in the identification of “causal” evidence for altered expression for genes within this locus. Most of these genes have not been associated with JIA before, and are involved in immune functions, including antigen processing, presentation, and cytokine signalling. Given the established associations between JIA and various diseases and traits, we hypothesized that the regulation of JIA causal genes and their interacting proteins could serve as biological connections to these traits. To investigate this, we integrated data from the blood GRN, PPIN, and GWAS, to identify 95 traits intersecting with JIA. Many of these traits had previously reported associations with JIA (Table 2). Furthermore, our approach pinpointed genes whose genetically regulated expression form the intersection between JIA and these associated traits. Overall, our findings deepen the understanding of the underlying biological mechanisms governing JIA and its associated traits, offering candidates for new biomarker discovery and shared therapeutic targets for patients with multimorbidity.

This study has several limitations. Firstly, MR depends on three key assumptions: the instrumental variables (spatial eQTLs) must be robustly associated with the exposure (gene expression); they should be free from confounders; and their influence on outcomes must occur solely through the exposure (i.e., no horizontal pleiotropy). We selected significant spatial eQTLs within the blood GRN (adjusted *p*-value ≤ 0.05; see methods) as instruments, likely fulfilling the first assumption. Verifying the second and third assumptions is challenging, but the use of randomly allocated genetic variants as instruments should naturally mitigate confounder effects (Smith and Ebrahim, 2004; Davey Smith and Hemani, 2014). Furthermore, several sensitivity analyses, including the Cochran Q statistic and MR-Egger (see methods; Supplementary Table S3), helped to remove exposures with potential horizontal pleiotropy (Burgess et al., 2023), reducing biases and errors in the downstream analyses. Secondly, our method to identify JIA-associated traits has its limitations: 1) the identified traits could be biased towards common traits that have been studied by GWAS; 2) any regulatory connections associated with genes without known PPIs in STRING (Szklarczyk et al., 2021) would be missed; 3) the identified traits should not be solely construed as having a positive association with JIA (such as comorbidity); negative associations (implying a protective effect) are also possible, as our approach did not account for the specific risk allele associated with the GWAS SNPs for each trait. Lastly, our analysis centered on the expanded PPI network within the blood GRN. However, we acknowledge that genetic variation might influence JIA risk through other cells or tissues, such as muscle, adipose, lung, or synovial fibroblast (Pudjihartono et al., 2023). Thus, future studies should incorporate GRNs from other tissues to obtain a more complete picture of the JIA disease process. Despite these limitations, our approach represents a step forward in understanding how genetic variations contribute to JIA and connect traits that intersect with JIA through shared molecular pathways.

Of the 52 causal genes identified, seven belong to the HLA class I or II gene family. HLA genes are integral to immune system regulation as they encode cell surface proteins, which are crucial for presenting self and foreign antigens to T-cells. Studies consistently rank genetic variations within the HLA genes as major contributors to the susceptibility of various autoimmune diseases (Simmonds and Gough, 2009), including JIA (Hersh and Prahalad, 2015; Hou et al., 2020). Moreover, dysregulation of HLA class I and II gene expression has been reported in the affected joints (Haas et al., 2009) and immune cells (Prigione et al., 2011; Imbach et al., 2023) of JIA patients. Notably, even after excluding the seven HLA genes from our functional enrichment analysis, there was still enrichment in immune-related terms (e.g., cytokine signalling pathways). Thus, our observations provide additional evidence for the involvement of the immune system in JIA, particularly in relation to aberrant antigen presentation and cytokine signalling.

Among causal genes involved in cytokine signaling, *LTA* (lymphotoxin-alpha or LTα) and *LTB* (lymphotoxin-beta or LTβ) stand out as they encode proteins that belong to the tumor necrosis factor (TNF) cytokine superfamily. LTα and LTβ interact to form a membrane-anchored heterotrimeric complex called the LTα1β2, which binds to and activates lymphotoxin-beta receptors (LTβR) (Bauer et al., 2012). Upon activation, LTβR initiates downstream signalling pathways resulting in the release of pro-inflammatory cytokines and chemokines (Nakano et al., 1996; Degli-Esposti et al., 1997; Chang et al., 2002; Piao et al., 2021). Interestingly, our MR results identified divergent gene-regulatory patterns for *LTA* and *LTB* as risks for JIA, with *LTA* being upregulated and *LTB* being downregulated (Table 1; Supplementary Table S2). We propose that downregulation of *LTB* may allow for more LTα proteins to exist in a soluble form (i.e., not associated with membrane-bound LTβ). LTα shares a structural similarity with TNFα, in its soluble form LTα exhibits a high affinity for binding to both TNF receptors 1 and 2 (TNFR1 and TNFR2) (Medvedev et al., 1996). Importantly, the functional ability of LTα to induce chemokine secretion and inflammatory gene expression through TNFR1 may be more potent than the effects of the LTα1β2 complex through LTβR (Williams-Abbott et al., 1997). Therefore, our results support the hypothesis that blocking LTα may serve as a viable target for JIA treatment, echoing previous suggestions for rheumatoid arthritis management (Buch et al., 2004; Calmon-Hamaty et al., 2011a; Calmon-Hamaty et al., 2011b). Likewise, specific inhibitors of TNFR1 could be useful to treat autoimmune diseases including JIA (Zhang et al., 2020).

Disease-associated gene regulation can be cell type-specific and does not uniformly affect an entire heterogeneous tissue (GTEx ConsortiumLaboratory Data Analysis &Coordinating Center LDACC—Analysis Working GroupStatistical Methods groups—Analysis Working GroupEnhancing GTEx eGTEx groupsNIH Common FundNIH/NCI et al., 2017). Thus, we sought to examine the gene-regulatory impacts of the SNPs that regulate the causal genes using an immune cell-type specific eQTL database (Schmiedel et al., 2018). Our analysis unveiled both shared and cell type-specific regulatory patterns amongst the causal genes (Figure 3). We propose that causal genes regulated across multiple immune cell types could contribute to JIA by influencing patients’ systemic inflammation profiles. For instance, *ERAP2* was upregulated by rs2927608 across all immune cell types. *ERAP2* encodes an intracellular enzyme responsible for trimming endogenous (self) proteins before presentation on HLA class I (de Castro and Stratikos, 2019). The upregulation of *ERAP2* might cause over-trimming of endogenous antigen (Mpakali et al., 2015; Venema et al., 2021), possibly leading to the production of antigens recognized by autoreactive CD8^+^ T cells (Pudjihartono et al., 2023). *BAK1*, a pro-apoptotic member of the BCL-2 protein family, was downregulated by rs210142 in all immune cell types. Apoptosis plays a crucial role in counteracting autoimmunity by maintaining cell counts and eliminating autoreactive immune cells (Chervonsky, 1999; Hutcheson et al., 2008; Croker et al., 2011). Indeed, disruption of the *BAK1*-mediated homeostatic mechanism in immune cells has been demonstrated to result in autoimmunity in mice upon *BAK1* deletion (Takeuchi et al., 2005). Conversely, genes with cell type-specific regulatory effects provide insights into the contributions of specific cell types to JIA aetiology. For example, *PPP1R11* was specifically downregulated by rs165256 in activated CD4^+^ T cells. *PPP1R11* encodes an inhibitor for protein phosphatase 1 (PP1) (Zhang et al., 1998), a pro-inflammatory protein whose activity augments activation-induced cytokine expression in CD4^+^ T cells (Mock, 2012). Indeed, silencing *PPP1R11* in CD4^+^ T cells renders them resistant to regulatory T cell-mediated suppression (Joshi et al., 2019). Therefore, our observations suggest that targeting the *PPP1R11* pathway in CD4^+^ T cells may modulate T cell activation and thus may be of therapeutic potential in JIA patients.

Our analysis revealed that JIA causal genes (level 0) and their interacting proteins (levels 1–2) are regulated by spatial eQTLs significantly enriched for 95 other GWAS traits. This suggests that these traits could be connected to JIA through the dysregulation of common biological pathways. Many of these traits have known associations with JIA (Table 2). However, we also identified traits with less clear connections to JIA, including platelet-to-lymphocyte ratio, sphingomyelin levels, cathepsin B levels, LILRB2 protein levels, multiple sclerosis, and giant cell arteritis. Interestingly, traits like platelet-to-lymphocyte ratio (Milovanovic et al., 2004; Zha et al., 2006; Fu et al., 2015; Gasparyan et al., 2019), sphingomyelin (Beckmann et al., 2017), cathepsin B (Ikeda et al., 2000; Behl et al., 2022), and LILRB2 protein levels (Huynh et al., 2007) have been positively associated with rheumatoid arthritis. These novel associations warrant further exploration in population-based studies, as they could serve as potential biomarkers for JIA. Additionally, identifying previously unknown disease-related traits, such as multiple sclerosis and giant cell arteritis, could inform a more comprehensive, multidisciplinary approach to managing JIA patients.

Through hierarchical clustering of gene-trait associations, we discovered clusters of pleiotropic genes (in level 0) whose genetically-regulated expression might underpin the connection between JIA and specific trait groups. Notably, two HLA class II genes (*HLA-DQB2, HLA-DRB1*) form the major links between JIA and most level 0 traits. This finding aligns with the highly pleiotropic nature of HLA genes, which has been implicated in various autoimmune diseases, and cancers (Dehaghani et al., 2002; Simmonds and Gough, 2009; Goebel et al., 2017; Zhong et al., 2019; Tamaki et al., 2021). Furthermore, we identified a group of genes located within chromosome 6p22.1 (*HLA-A, HCG4P5, HLA-T, MOG, TRIM26, HCG9, IFITM4P*; cluster 5) that underlies the association between JIA and a specific subset of traits. This subset includes autoimmune/inflammatory traits such as type 1 diabetes, asthma, eczema, severe COVID-19, and rheumatoid arthritis, in addition to other traits (e.g., platelet-to-lymphocyte ratio, IgE level, and shingles). Importantly, the known functions of these genes may provide valuable insights into the biological connections between JIA and autoimmune/inflammatory diseases. For example, *TRIM26* encodes an E3 ubiquitin ligase that negatively regulates innate antiviral responses and the production of type 1 interferon (Shrivastav and Niewold, 2013; Wang et al., 2015). *IFITM4P* has also been shown as a positive regulator of innate antiviral immunity (Xiao et al., 2021). Defective innate antiviral responses could predispose individuals to autoimmunity or trigger a flare of an existing autoimmune disease (Stergioti et al., 2022).

JIA patients are at elevated risk of developing lymphoproliferative (e.g., lymphoma, leukemia) and other cancers (Nordstrom et al., 2012; Horne et al., 2019b). However, the reasons behind the development of malignancies in autoimmune patients are still debated. On one hand, certain immunosuppressive drugs to treat autoimmune diseases may weaken the immune system for eliminating cancer cells, consequently increasing cancer risk (Kinlen, 1985). Alternatively, there may be shared biological mechanisms between autoimmunity and certain types of cancers. For example, chronic inflammation, persistent cytokine and chemokine presence, and the activation of key transcription factors (e.g., NF-κB) can contribute to DNA instability and mutations (Hussain and Harris, 2007; Moore et al., 2010). Additionally, both autoimmunity and lymphoproliferative cancers manifest shared characteristics (e.g., heightened lymphocyte proliferation) (Dameshek and Schwartz, 1959; Edward, 2018). Our analysis identified several types of carcinomas and lymphoproliferative cancers as associated with JIA (in level 0). Notably, in addition to HLA class II genes (which might contribute to chronic inflammation), we found a distinct association between JIA and Hodgkin lymphoma through a set of genes in 6p21.3 (*FKBPL, PBX2, AGER*); and chronic lymphocytic leukaemia through the *BAK1* gene. *BAK1* is known for its role in regulating apoptosis, and *PBX2* encodes a transcription factor that influences cell proliferation and differentiation (Kamps et al., 1991; Phelan et al., 1995; Qiu et al., 2009). Consequently, abnormal expression of *BAK1* and *PBX2* may contribute to uncontrolled lymphocyte proliferation. This hypothesis aligns with observations in mice, where dysfunctional apoptosis regulation (via *FAS* mutation) resulted in both autoimmune disease and lymphoma (Rieux-Laucat et al., 1995; Straus et al., 2001). We also noted an association between JIA and the platelet-to-lymphocyte ratio mediated through the *BAK1* gene. Interestingly, mice lacking *BAK1* have previously been shown to exhibit increased circulating platelet lifespan (Mason et al., 2007; Josefsson et al., 2011).

Although population studies indicate that up to 7% of JIA patients fall into the “juvenile psoriatic arthritis” (JPsA) category, the gene-regulatory mechanisms underlying this condition remain unclear (Consolaro et al., 2019). Our analysis, however, uncovered that proteins encoded by ten genes (*MAPT, HLA-B, HLA-C, HLA-F, HLA-DQA2, HLA-DOB, TAP2, PSMB9, TYK2, MICB*) regulated by spatial eQTLs associated with psoriatic arthritis, directly interact with proteins encoded by nine JIA causal genes. These psoriatic arthritis-associated genes are involved in various cellular pathways, ranging from antigen processing and presentation to immune cell activation, and cytokine signaling (Figure 6B). Interestingly, some of these genes (e.g., *HLA-C, HLA-B, TAP2, MAPT,* and *TYK2*) have been previously implicated in the pathogenesis of psoriasis (Carlén et al., 2007; Prinz, 2018; Cai et al., 2020; Baran et al., 2022; Shang et al., 2022). We propose that the protein-protein interactions we identified contribute to a common pathway disruption, potentially leading to psoriatic symptoms in a subset of JIA patients.

Overall, our findings have provided insights into the biological mechanisms underpinning JIA pathogenesis and its connections with other traits. We have discussed some of the biological rationale underlying JIA causal genes and their potential links to other traits. However, not all discovered genes and connections have experimentally-known functions, leaving their roles in the JIA disease process open to further investigation. Our results suggest that the burden of associated traits may differ among JIA patients, influenced by their combined genetic risk across different clusters of traits. We argue that future experimental validation of these connections could pave the way for refined patient stratification, the discovery of new biomarkers, and the discovery of shared therapeutic targets.

## Data Availability

Datasets generated and utilized during this research can be found in the supplementary tables or in online repositories (names and accession numbers are provided in the article). The scripts used for data analysis and figure creation are available at: https://github.com/nicholaspudjihartono/JIA_Associated_Traits/.

## Supplementary material

SUPPLEMENTARY FIGURE S1 Venn diagram of JIA-associated genes from DisGeNET, GWAS catalog “nearest genes”, and two-sample MR.

SUPPLEMENTARY FIGURE S2 Hierarchical clustering of gene-trait associations occurring at level 2 of the JIA PPIN. Clustering was based on the number of GWAS SNPs that overlapped (or in LD r2 ≥ 0.8 with) spatial eQTLs regulating level 2 genes. For simplicity, all genes (columns) with only one trait association were removed. See Supplementary Table S14 for raw data.
