# Supplementary figures and images for: Integrative analysis reveals novel insights into juvenile idiopathic arthritis pathogenesis and shared molecular pathways with associated traits
Source: Front Genet. 2024 Aug 8;15:1448363. doi: 10.3389/fgene.2024.1448363 (PMC11338781; doi:10.3389/fgene.2024.1448363)

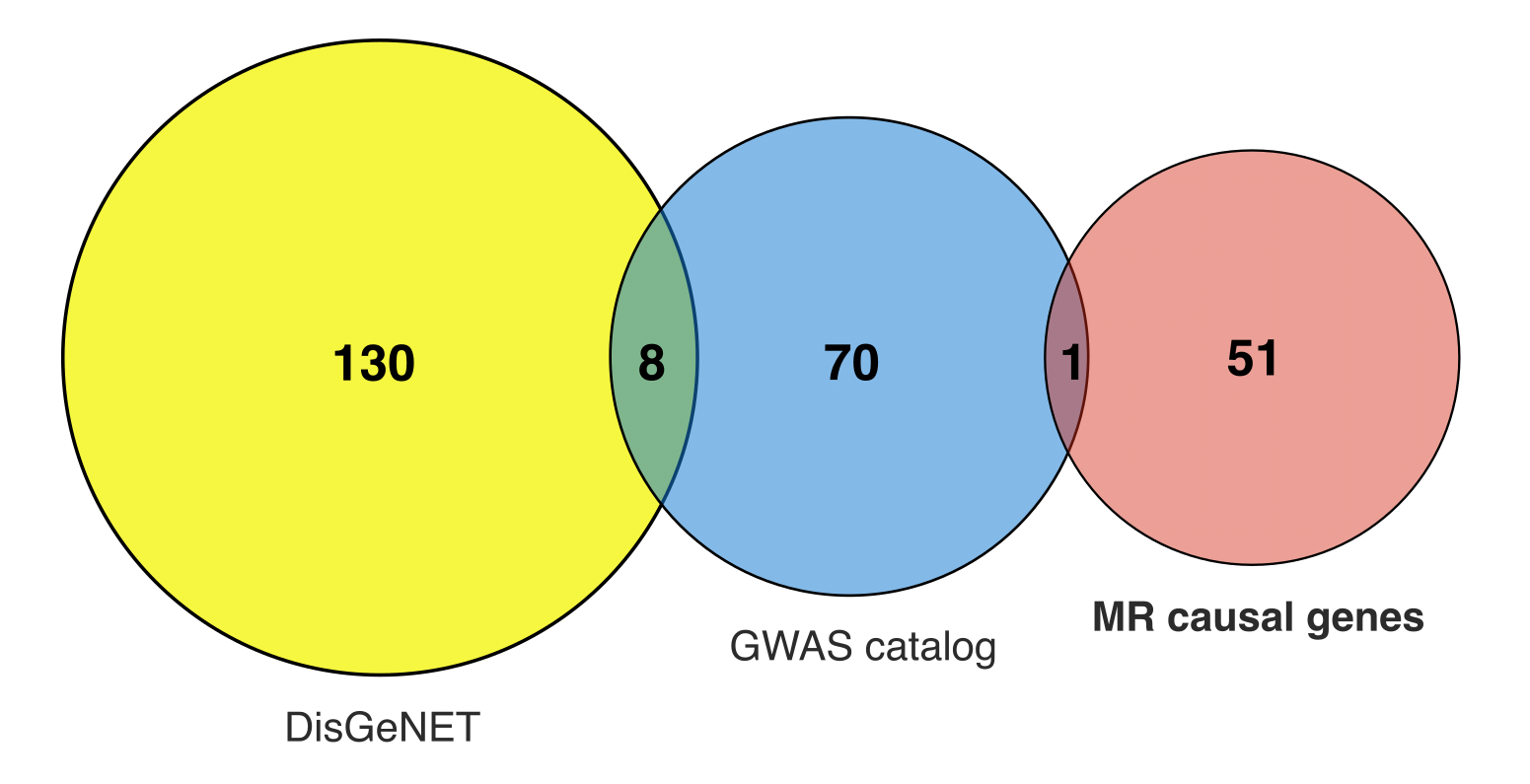

Supplement: Supplementary file 1 [file Image1.TIFF]

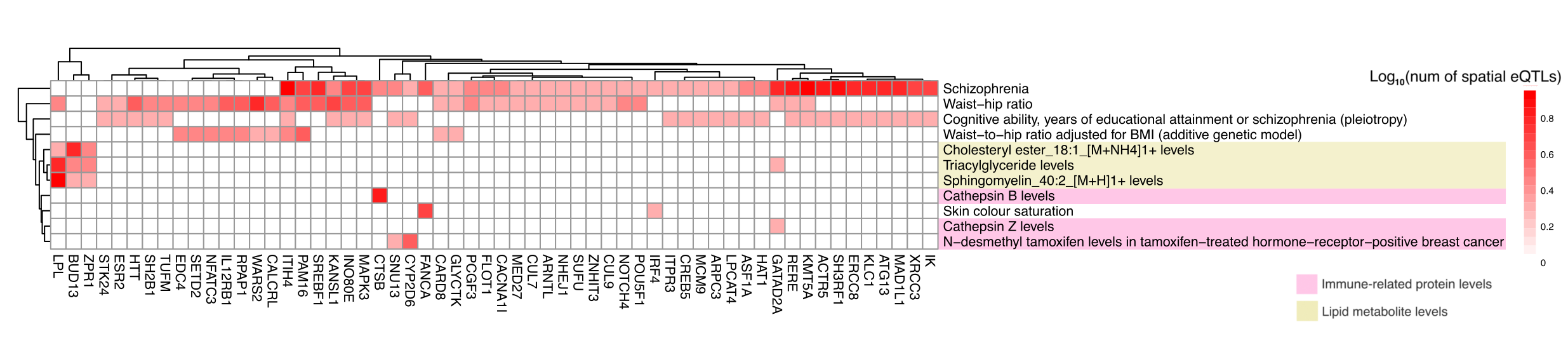

Supplement: Supplementary file 3 [file Image2.TIFF]
