# Supplementary material for: Integrative analysis reveals novel insights into juvenile idiopathic arthritis pathogenesis and shared molecular pathways with associated traits
Source: Front Genet. 2024 Aug 8;15:1448363. doi: 10.3389/fgene.2024.1448363 (PMC11338781; doi:10.3389/fgene.2024.1448363)
Supplement: Supplementary file 4 [file DataSheet1.DOCX]

**STROBE-MR checklist of recommended items to address in reports of Mendelian randomization studies**^1^ ^2^

| **Item No.** | **Section** | **Checklist item** | **Page No.** | **Relevant text from manuscript** |
| --- | --- | --- | --- | --- |
| 1 | **TITLE and ABSTRACT** | Indicate Mendelian randomization (MR) as the study’s design in the title and/or the abstract if that is a main purpose of the study | Detailed in abstract and introduction section | In this study, we performed two-sample MR using spatial eQTLs within the blood gene regulatory network (GRN) as instrumental variables to identify 52 potential JIA causal genes |
|  | **INTRODUCTION** |  |  |  |
| 2 | **Background** | Explain the scientific background and rationale for the reported study. What is the exposure? Is a potential causal relationship between exposure and outcome plausible? Justify why MR is a helpful method to address the study question | Detailed in introduction section | The majority of the genetic loci associated with JIA are located in the non-coding regions of the genome. Studies have demonstrated that disease-associated variants (e.g., Single nucleotide polymorphisms [SNPs]) are enriched within regulatory DNA elements. One of the key mechanisms of gene regulation involves direct physical interaction between distal regulatory elements and their target genes. Previous work by our group has investigated how JIA-associated SNPs influence the expression of distant genes through SNP-gene physical interaction. |
| 3 | **Objectives** | State specific objectives clearly, including pre-specified causal hypotheses (if any). State that MR is a method that, under specific assumptions, intends to estimate causal effects | Detailed in introduction section | We contend that by using variants that physically interact and are associated with gene expression (termed ‘spatial eQTL SNPs’) as instrumental variables and GWAS data to asses its impact to disease outcome, we can utilize MR to infer a causal relationship between altered gene expression and specific disease outcomes. |
|  | **METHODS** |  |  |  |
| 4 | **Study design and data sources** | Present key elements of the study design early in the article. Consider including a table listing sources of data for all phases of the study. For each data source contributing to the analysis, describe the following: |  |  |
|  | a) | Setting: Describe the study design and the underlying population, if possible. Describe the setting, locations, and relevant dates, including periods of recruitment, exposure, follow-up, and data collection, when available. | Detailed in methods section | The blood GRN was constructed by identifying spatial eQTLs involving all common SNPs (MAF ≥ 0.05; n=~40x10^6) present within the whole blood eQTL dataset (from Genotype – Tissue Expression Project [GTEx V8])  *NOTE : Detailed information and study design of the GTEx whole blood eQTL data can be found in the cited GTEx publication* ^3^*.*  A recent JIA GWAS by López-Isac et al. was selected for the outcome data due to its large sample size, comprising 3,305 JIA cases and 9,196 healthy controls (https://www.ebi.ac.uk/ gwas/ downloads/ summary- statistics) (Study Accession Code : GCST90010715)  *NOTE : Detailed information and study design of the JIA GWAS can be found in the cited JIA GWAS publication* ^4^*.* |
|  | b) | Participants: Give the eligibility criteria, and the sources and methods of selection of participants. Report the sample size, and whether any power or sample size calculations were carried out prior to the main analysis |  | *NOTE : Detailed information and study design of the GTEx whole blood eQTL data can be found in the cited GTEx publication* ^3^*.*  *NOTE : Detailed information and study design of the JIA GWAS can be found in the cited JIA GWAS publication* ^4^*.* |
|  | c) | Describe measurement, quality control and selection of genetic variants |  | *NOTE : Detailed information and study design of the GTEx whole blood eQTL data can be found in the cited GTEx publication* ^3^*.*  *NOTE : Detailed information and study design of the JIA GWAS can be found in the cited JIA GWAS publication* ^4^*.* |
|  | d) | For each exposure, outcome, and other relevant variables, describe methods of assessment and diagnostic criteria for diseases |  | *NOTE : Detailed information and study design of the JIA GWAS can be found in the cited JIA GWAS publication* ^4^*.* |
|  | e) | Provide details of ethics committee approval and participant informed consent, if relevant |  | *NOTE : Detailed information and study design of the GTEx whole blood eQTL data can be found in the cited GTEx publication* ^3^*.*  *NOTE : Detailed information and study design of the JIA GWAS can be found in the cited JIA GWAS publication* ^4^*.* |
| 5 | **Assumptions** | Explicitly state the three core IV assumptions for the main analysis (relevance, independence and exclusion restriction) as well assumptions for any additional or sensitivity analysis | Detailed in methods section | MR analysis relies on three main assumptions: First, the genetic instruments (i.e., spatial eQTLs) must be robustly associated with the exposure of interest (i.e., gene expression). Second, these instruments should be independent of any potential confounders. Third, the genetic instruments influence the outcomes solely through their association with the exposure. |
| 6 | **Statistical methods: main analysis** | Describe statistical methods and statistics used |  |  |
|  | a) | Describe how quantitative variables were handled in the analyses (i.e., scale, units, model) | - | - |
|  | b) | Describe how genetic variants were handled in the analyses and, if applicable, how their weights were selected | Detailed in methods section | We used only statistically significant spatial eQTL-gene pairs within the blood GRN (adjusted p-value ≤0.05) as exposure instruments. Furthermore, to ensure that instrumental variables for each exposure was independent, we performed LD clumping with r^2^ cutoff of 0.001. For this, the European (EUR) population from the 1000 Genomes project served as the reference panel for LD analysis. |
|  | c) | Describe the MR estimator (e.g. two-stage least squares, Wald ratio) and related statistics. Detail the included covariates and, in case of two-sample MR, whether the same covariate set was used for adjustment in the two samples | Detailed in methods section | After harmonizing the exposure and outcome data, genes with one spatial eQTL associated with it underwent 2SMR using the Wald test, whereas those with two spatial eQTLs underwent two-sample MR using the inverse variance weighted method, those with 3 or more spatial eQTLs underwent two-sample MR using inverse variance weighted and weighted median methods. Genes whose *p*-value was equal to or below the Bonferroni-corrected threshold (0.05/number of unique exposure genes [13,640]) were considered statistically significant two-sample MR results and therefore have a putatively causal role in JIA within the whole blood tissue. |
|  | d) | Explain how missing data were addressed | - | - |
|  | e) | If applicable, indicate how multiple testing was addressed | Detailed in methods section | Genes whose *p*-value was equal to or below the Bonferroni-corrected threshold (0.05/number of unique exposure genes [13,640]) were considered statistically significant two-sample MR results and therefore have a putatively causal role in JIA within the whole blood tissue. |
| 7 | **Assessment of assumptions** | Describe any methods or prior knowledge used to assess the assumptions or justify their validity | Detailed in methods section | To satisfy the first assumption, we used only statistically significant spatial eQTL-gene pairs within the blood GRN (adjusted p-value ≤0.05) as exposure instruments. |
| 8 | **Sensitivity analyses and additional analyses** | Describe any sensitivity analyses or additional analyses performed (e.g. comparison of effect estimates from different approaches, independent replication, bias analytic techniques, validation of instruments, simulations) | Detailed in methods section | For genes with two or more instrumental variables, several sensitivity analyses were conducted to identify the presence of horizontal pleiotropy. Cochran's Q was computed to quantify the variation in causal effect estimates attributed to different genetic instruments. A *p*-value ≤ 0.05 suggests significant heterogeneity, which may indicate pleiotropy or other issues such as invalid instrumental variables. Furthermore, we performed an MR-Egger regression to test for horizontal pleiotropy by evaluating its intercept. A significant non-zero intercept (*p*-value ≤ 0.05) is considered evidence of horizontal pleiotropy. Exposure genes that failed to pass these sensitivity analyses were removed from the final causal gene list. |
| 9 | **Software and pre-registration** |  |  |  |
|  | a) | Name statistical software and package(s), including version and settings used | Detailed in methods section | To identify potentially causal genes for JIA within the blood GRN, we conducted a two-sample Mendelian Randomization using the TwoSampleMR R package (<https://github.com/MRCIEU/TwoSampleMR/>, version 0.5.6) |
|  | b) | State whether the study protocol and details were pre-registered (as well as when and where) | - | - |
|  | **RESULTS** |  |  |  |
| 10 | **Descriptive data** |  |  |  |
|  | a) | Report the numbers of individuals at each stage of included studies and reasons for exclusion. Consider use of a flow diagram | Detailed in methods section | *NOTE : Detailed information and study design of the GTEx whole blood eQTL data can be found in the cited GTEx publication* ^3^*.*  *NOTE : Detailed information and study design of the JIA GWAS can be found in the cited JIA GWAS publication* ^4^ |
|  | b) | Report summary statistics for phenotypic exposure(s), outcome(s), and other relevant variables (e.g. means, SDs, proportions) | Detailed in the results and methods section | *NOTE : Summary statistics for the JIA GWAS can be accessed from the original source (https://www.ebi.ac.uk/ gwas/ downloads/ summary- statistics) (Study Accession Code : GCST90010715)*  *NOTE : A comprehensive description of the blood GRN have been previously published* ^5^*.* |
|  | c) | If the data sources include meta-analyses of previous studies, provide the assessments of heterogeneity across these studies | - | - |
|  | d) | For two-sample MR:  i.  Provide justification of the similarity of the genetic variant-exposure associations between the exposure and outcome samples  ii.  Provide information on the number of individuals who overlap between the exposure and outcome studies |  | *NOTE : Detailed information and study design of the GTEx whole blood eQTL data can be found in the cited GTEx publication* ^3^*^.^*  *NOTE : Detailed information and study design of the JIA GWAS can be found in the cited JIA GWAS publication* ^3^ |
| 11 | **Main results** |  |  |  |
|  | a) | Report the associations between genetic variant and exposure, and between genetic variant and outcome, preferably on an interpretable scale |  | Table S11 |
|  | b) | Report MR estimates of the relationship between exposure and outcome, and the measures of uncertainty from the MR analysis, on an interpretable scale, such as odds ratio or relative risk per SD difference |  | Table S2 |
|  | c) | If relevant, consider translating estimates of relative risk into absolute risk for a meaningful time period | - | - |
|  | d) | Consider plots to visualize results (e.g. forest plot, scatterplot of associations between genetic variants and outcome versus between genetic variants and exposure) | - | - |
| 12 | **Assessment of assumptions** |  |  |  |
|  | a) | Report the assessment of the validity of the assumptions | Detailed in methods section and supplementary tables | To satisfy the first assumption, we used only statistically significant spatial eQTL-gene pairs within the blood GRN (adjusted p-value ≤0.05) as exposure instruments.  *NOTE : The result of sensitivity analyses are reported in Table S12* |
|  | b) | Report any additional statistics (e.g., assessments of heterogeneity across genetic variants, such as *I^2^*, Q statistic or E-value) |  | Table S12 |
| 13 | **Sensitivity analyses and additional analyses** |  |  |  |
|  | a) | Report any sensitivity analyses to assess the robustness of the main results to violations of the assumptions |  | Table S12 |
|  | b) | Report results from other sensitivity analyses or additional analyses |  | Table S12 |
|  | c) | Report any assessment of direction of causal relationship (e.g., bidirectional MR) | - | - |
|  | d) | When relevant, report and compare with estimates from non-MR analyses | - | - |
|  | e) | Consider additional plots to visualize results (e.g., leave-one-out analyses) | - | - |
|  | **DISCUSSION** |  |  |  |
| 14 | **Key results** | Summarize key results with reference to study objectives | Detailed in discussion section | In this study, we conducted a two-sample MR analysis on JIA by integrating large-scale GWAS and spatial eQTL data obtained from whole blood samples (i.e., blood GRN). This approach enables the evaluation of causal associations between changes in gene expression and JIA outcomes (Figure 1b). Our analysis identified 52 genes that have potential causal roles in JIA. These genes have not been associated with JIA before, and are involved in immune functions, including antigen processing, presentation, and cytokine signalling. Notably, some of the IV SNPs associated with causal genes exhibit immune cell type-specificity, offering valuable insights into their contributions to JIA pathogenesis. |
| 15 | **Limitations** | Discuss limitations of the study, taking into account the validity of the IV assumptions, other sources of potential bias, and imprecision. Discuss both direction and magnitude of any potential bias and any efforts to address them | Detailed in discussion section | This study has several limitations. Firstly, MR depends on three key assumptions: the instrumental variables (spatial eQTLs) must be robustly associated with the exposure (gene expression); they should be free from confounders; and their influence on outcomes must occur solely through the exposure (i.e., no horizontal pleiotropy). We selected significant spatial eQTLs within the blood GRN (adjusted *p*-value ≤0.05; see methods) as instruments, likely fulfilling the first assumption. Verifying the second and third assumptions is challenging, but the use of randomly allocated genetic variants as instruments should naturally mitigate confounder effects . Furthermore, several sensitivity analyses, including the Cochran Q statistic and MR-Egger (see methods; Table S12), helped to remove exposures with potential horizontal pleiotropy , reducing biases and errors in the downstream analyses. |
| 16 | **Interpretation** |  |  |  |
|  | a) | Meaning: Give a cautious overall interpretation of results in the context of their limitations and in comparison with other studies | - | - |
|  | b) | Mechanism: Discuss underlying biological mechanisms that could drive a potential causal relationship between the investigated exposure and the outcome, and whether the gene-environment equivalence assumption is reasonable. Use causal language carefully, clarifying that IV estimates may provide causal effects only under certain assumptions | Detailed in discussion section | Of the 52 causal genes identified, nine belong to the HLA class I or II gene family. The highly polymorphic HLA genes are integral to immune system regulation as they encode cell surface proteins, which are crucial for presenting self and foreign antigens to T-cells. Studies consistently rank genetic variations within the HLA region as major contributors to the susceptibility of various autoimmune diseases, including JIA. Moreover, dysregulation of HLA gene expression has been reported in the affected joints and immune cells of JIA patients. However, the HLA region is estimated to explain ~8–13% of JIA’s total heritability, suggesting that non-HLA genes make crucial contributions . Notably, even after excluding the nine HLA genes from our functional enrichment analysis, there was still enrichment in immune-related terms (e.g. cytokine signalling pathways). Thus, our observations provide additional evidence for the involvement of the immune system in JIA, particularly in relation to aberrant antigen presentation and cytokine signalling.  Among causal genes involved in cytokine signaling, LTA (lymphotoxin-alpha or LTα) and LTB (lymphotoxin-beta or LTβ) stand out as they encode proteins that belong to the tumor necrosis factor (TNF) cytokine superfamily. LTα and LTβ interact to form a membrane-anchored heterotrimeric complex called the LTα1β2, which binds to and activates lymphotoxin-beta receptor (LTβR). Upon activation, LTβR initiates downstream signalling pathways resulting in the release of pro-inflammatory cytokines and chemokines. Interestingly, our MR results identified divergent gene-regulatory patterns for LTA and LTB as risk for JIA, with LTA being up-regulated and LTB being down-regulated (Table 1 and Table S2). We propose that down-regulation of LTB may allow for more LTα proteins to exist in a soluble form (i.e. not associated with membrane-bound LTβ). LTα shares a structural similarity with TNFα, in its soluble form LTα exhibits a high affinity for binding to both TNF receptors 1 and 2 (TNFR1 and TNFR2). Importantly, the functional ability of LTα to induce chemokine secretion and inflammatory gene expression through TNFR1 may be more potent than the effects of the LTα1β2 complex through LTβR. Therefore, our results support the hypothesis that blocking LTα may serve as a viable target for JIA treatment, echoing previous suggestions for rheumatoid arthritis management. Likewise, specific inhibitors of TNFR1 could be useful to treat autoimmune diseases including JIA |
|  | c) | Clinical relevance: Discuss whether the results have clinical or public policy relevance, and to what extent they inform effect sizes of possible interventions | Detailed in discussion section | See above |
| 17 | **Generalizability** | Discuss the generalizability of the study results (a) to other populations, (b) across other exposure periods/timings, and (c) across other levels of exposure | - | - |
|  | **OTHER INFORMATION** |  |  |  |
| 18 | **Funding** | Describe sources of funding and the role of funders in the present study and, if applicable, sources of funding for the databases and original study or studies on which the present study is based | Detailed in acknowledgment and funding section | The authors would like to acknowledge the Dines Family Trust and Wellcome Leap (M4EFaD, 3725046) for their donations that supported the work of JOS and DH. NP received the University of Auckland PhD scholarship. |
| 19 | **Data and data sharing** | Provide the data used to perform all analyses or report where and how the data can be accessed, and reference these sources in the article. Provide the statistical code needed to reproduce the results in the article, or report whether the code is publicly accessible and if so, where | Detailed in data and code availability section | Data analyses and visualizations were performed in Python (version 3.8.12) through Jupyter notebook (version 6.4.6) or using R (version 4.0.4) through RStudio (version 1.4.1106). Datasets generated and utilized during this research are provided in the supplementary tables. The scripts utilized for data analysis and the creation of figures are available on GitHub (https://github.com/nicholaspudjihartono/JIA_Associated_Traits/). |
| 20 | **Conflicts of Interest** | All authors should declare all potential conflicts of interest |  | The authors declare that they have no competing interests. |

This checklist is copyrighted by the Equator Network under the Creative Commons Attribution 3.0 Unported (CC BY 3.0) license.

1. Skrivankova VW, Richmond RC, Woolf BAR, Yarmolinsky J, Davies NM, Swanson SA, et al. Strengthening the Reporting of Observational Studies in Epidemiology using Mendelian Randomization (STROBE-MR) Statement. JAMA. 2021;under review.

2. Skrivankova VW, Richmond RC, Woolf BAR, Davies NM, Swanson SA, VanderWeele TJ, et al. Strengthening the Reporting of Observational Studies in Epidemiology using Mendelian Randomisation (STROBE-MR): Explanation and Elaboration. BMJ. 2021;375:n2233.

3. Aguet F, Barbeira AN, Bonazzola R, Brown A, Castel SE, Jo B, et al. The GTEx Consortium atlas of genetic regulatory effects across human tissues. Science (1979). 2020 Sep 1;369(6509):1318–30.

4. López-Isac E, Smith SL, Marion MC, Wood A, Sudman M, Yarwood A, et al. Combined genetic analysis of juvenile idiopathic arthritis clinical subtypes identifies novel risk loci, target genes and key regulatory mechanisms. Ann Rheum Dis [Internet]. 2021 [cited 2021 Sep 1];80(3):321–8. Available from: https://ard.bmj.com/content/80/3/321.abstract

5. Zaied RE, Fadason T, O’Sullivan JM. De novo identification of complex traits associated with asthma. Front Immunol. 2023 Aug 23;14.
